# The Evolution of an Interprofessional Shared Decision-Making Research Program: Reflective Case Study of an Emerging Paradigm

**DOI:** 10.5334/ijic.2212

**Published:** 2016-07-19

**Authors:** Maman Joyce Dogba, Matthew Menear, Dawn Stacey, Nathalie Brière, France Légaré

**Affiliations:** 1 Department of Family and Emergency Medicine, Université Laval, Quebec, Canada; 2 Research Centre of the CHU de Québec-Université Laval, Québec, Canada; 3 Faculty of Health Sciences, School of Nursing, University of Ottawa, Ottawa, Ontario, Canada; 4 Ottawa Hospital Research Institute, Ottawa, Ontario, Canada; 5 CIUSSS de la Capitale Nationale, Québec, Canada; 6 Canada Research Chair in Shared Decision Making and Knowledge Translation, Université Laval, Québec, Canada

**Keywords:** shared decision-making, interprofessional, primary care, knowledge construction, Kuhn’s theory, reflective case study

## Abstract

**Introduction:**

Healthcare research increasingly focuses on interprofessional collaboration and on shared decision making, but knowledge gaps remain about effective strategies for implementing interprofessional collaboration and shared decision-making together in clinical practice. We used Kuhn’s theory of scientific revolutions to reflect on how an integrated interprofessional shared decision-making approach was developed and implemented over time.

**Methods:**

In 2007, an interdisciplinary team initiated a new research program to promote the implementation of an interprofessional shared decision-making approach in clinical settings. For this reflective case study, two new team members analyzed the team’s four projects, six research publications, one unpublished and two published protocols and organized them into recognizable phases according to Kuhn’s theory.

**Results:**

The merging of two young disciplines led to challenges characteristic of emerging paradigms. Implementation of interprofessional shared-decision making was hindered by a lack of conceptual clarity, a dearth of theories and models, little methodological guidance, and insufficient evaluation instruments. The team developed a new model, identified new tools, and engaged knowledge users in a theory-based approach to implementation. However, several unresolved challenges remain.

**Discussion:**

This reflective case study sheds light on the evolution of interdisciplinary team science. It offers new approaches to implementing emerging knowledge in the clinical context.

## Introduction

Health service delivery is now characterized by an ethos of partnership, reflected by the growing emphasis on providing comprehensive services (e.g. through teamwork and interprofessional collaboration) and on active collaboration with patients, their families and caregivers, and communities (e.g. patient-centred care, shared decision-making, citizen advisory boards) [1,2,3]. Grounded in this emerging ethos of partnership, the fields of research on interprofessional collaboration for patient-centred care and shared decision-making in healthcare are each still relatively young [4,5]. Over the past decades there has been a shift in focus from the patient-doctor dyad as the primary unit of analysis to the interprofessional healthcare team [6]. At the same time, there has been a shift from the paternalistic, doctor-knows-best model of decision-making to the shared decision-making model. These models have been shown to produce better patient outcomes than their predecessors [7,8,9].

Historically, the fields of research on interprofessional collaboration and shared decision-making have evolved along separate tracks. Until the 1980s, the field of interprofessional collaboration was often plagued by a lack of conceptual and methodological clarity. It was not until the 1990s that new programs of research led to a more consistent advancement of knowledge in the field [5], followed in the 2000s by advances in theorizing and measurement of interprofessional collaboration. In spite of these advances, the specific role of patients within interprofessional teams has remained largely undefined [10]. In a similar vein, research on patient and family involvement in healthcare decision-making has largely ignored the increasingly interprofessional nature of healthcare delivery. Use of the term “shared decision-making” emerged in the early 1980s, although the foundations for research on this topic are based on earlier investigations in medical decision-making and patient-provider relationships. As with research on interprofessional collaboration, the shared decision-making field has seen many debates about definitions and theories of shared decision-making and about outcomes [11]. However, a review of the literature has shown little integration of the notion of interprofessional collaboration in conceptualizations of shared decision-making [12].

Merging the fields of interprofessional and shared decision-making has the potential to generate new knowledge about the delivery of more patient-centred forms of care in modern healthcare systems where more than half of care is delivered by interprofessional teams [3,13,14,15,16,17]. However, little is known about the challenges encountered in developing and conducting a research program centred on an interprofessional approach to shared decision-making. The aim of this paper therefore is to present a reflective case study account of an effort to develop an interprofessional shared decision-making (IP-SDM) research program led by an interdisciplinary research team from Canada. Our reflections were guided by three main questions: 1) what were the main challenges that the research team faced over the course of the research program? 2) How did the research team overcome these challenges? 3) What practical lessons can be drawn from their experience and what are the future directions for research on the interprofessional shared decision-making approach? We hope that the experience of our research team provides insights to others who are similarly confronted by the paradigmatic challenges that arise from interdisciplinary research.

## Methods

### Critical reflective approach

This reflective case study was led by two young researchers who, in 2014, joined an interdisciplinary research team working on clinical interventions in interprofessional shared decision-making. Both researchers had professional backgrounds in primary care and public health research and sought to become familiar with the IP-SDM approach and research program. During this process, they determined that a critical analysis of the overall research program could lead to novel insights for the team and inform their own research activities within the program. Based on her previous work, the first author proposed a theoretical framework to focus thinking about the research program, help analyze the foundations and evolution of the interprofessional shared decision-making approach, and draw conclusions about future directions for researchers interested in this approach. The young researchers also met with the larger research team to discuss preliminary findings and enrich their analysis.

### Theoretical framework

The theoretical framework supporting our reflective analysis was based on Thomas Kuhn’s theory of scientific revolutions [18]. Kuhn argued that scientific progress does not result from an incremental accumulation of individual discoveries and inventions, but rather through successive transitions from one dominant “paradigm” to another. The theory posits that paradigms are sets of beliefs that are accepted and shared by a community of scientists about the questions to be asked at a specific time and about the methods and approaches to answering these questions. However, as science progresses over time and knowledge is acquired, there are periods in which new questions emerge that challenge established paradigms. These “crises” or periods of transition prompt new exploration and speculation, and the development of new theories about observed phenomena, as well as debates about problems and solutions. Over time, a dominant paradigm emerges that more satisfactorily explains the evidence and replaces earlier frameworks. This in turn leads to a “normalization” of scientific work – that is, to the articulation of theories that are consistent with the new paradigm and that attempt to reconcile anomalies. In this phase, scientists narrow their vision of the field and undertake increasingly detailed work. The cycle of crisis, discovery, and normalization is repeated over time. Kuhn’s theory and concepts have received much attention and have been used to help make sense of how certain fields of scientific research evolve [19]. This theory helped us to identify four phases in which to chronologically chart the evolution of the research program 1) the stable period of the old paradigms, 2) the crises or questions that challenged the old paradigms, 3) the emergence of the new paradigm and 4) the normalization of the new paradigm and the solving of new puzzles.

In Kuhn’s model, scientific paradigms share four sets of rules or dimensions: conceptual, theoretical, methodological, and instrumental [20]. To be more consistent in our coding scheme, in our analysis of the emerging paradigm we defined these dimensions in the following ways [19]: 1) conceptual – agreed-on definitions of essential elements of interprofessional collaboration and shared decision-making and objects of study considered legitimate, 2) theoretical – the theories behind interprofessional collaboration and shared decision-making, and their relations to one another and to the world, 3) methodological – the established methods for investigating interprofessional collaboration and shared decision-making, and 4) instrumental – the accepted tools and techniques used to assess interprofessional collaboration and shared decision-making. Thus a matrix in which the columns comprise the four phases was used to reflect on the course of the research team towards a merged interprofessional *and* shared decision-making approach, and the four dimensions were used to identify the challenges associated with the second phase, i.e. the emergence of a new paradigm.

### Analytic approach and data sources

The foundation and evolution of the research team’s program was analyzed by gathering published articles and study protocols related to the team’s interprofessional shared decision-making research projects and by performing reference list searches in these documents. Identifying and reviewing the most commonly cited references provided us with an understanding of the main sources of knowledge on interprofessional collaboration and shared decision-making on which the projects were based [11,14,21,22,23]. The data were sorted chronologically to describe the research team’s activities over time, and an initial review of this literature allowed a long-time team member and the two new members to consensually define the questions and procedures for the analysis. The two new members then independently reviewed and analyzed the interprofessional shared decision-making research articles and study protocols using a method consistent with directed content analysis, a deductive form of textual analysis [24]. A data extraction matrix was created in Excel to systematically collect data on Kuhn’s stages and on the four dimensions of the “new paradigm”, as well as data on study authors, study and publication dates, stakeholders involved or targeted by the study, and type of study (e.g. review, theory-based study, methodological, descriptive, intervention). They then deepened their critical and reflective analysis by meeting four times with the three original members of the research team to review emerging findings, reflect on the origins of the research program and identify factors influencing its evolution. Finally, reverse citation searches of the team’s key publications within the research program were conducted to assess the program’s influence within the broader research community.

## Results

The search yielded six original research articles published by the research team, two published study protocols, and two unpublished study protocols used in grant applications. These documents stemmed from four funded projects (see Table 1). The reference lists of these articles and protocols allowed us to identify 224 unique references that informed the research program. The results of our reflective study are presented chronologically according to the four phases identified above.

**Table 1:** Project development: timeline of objectives, research, publications and citations.

Funded project	Objectives	Studies & key publications	Dates data collected and analysed											

			2007	2008	2009	2010	2011	2012	2013	2014	2015	2016	2017	2018
			
A) Advancing theories, models and measurement for an interprofessional approach to shared decision making in primary care	Develop conceptual model and propose measures	**Study protocol A1** *Advancing theories, models and measurement for an interprofessional approach to shared decision making in primary care: a study protocol.* BMC Health Serv Res, 2008. **8**(1)	✓	✓	✓	✓								
	Analyse models and determine their relevance to clinical practice	**Study A2** Stacey, D., et al., *Shared decision making models to inform an interprofessional perspective on decision making: a theory analysis.* Patient Educ Couns, 2010. **80**(2): p. 164–72.	✓	✓										
	Propose and validate a new model	**Study A3** Legare, F., et al., *An interprofessional approach to shared decision making: an exploratory case study with family caregivers of one IP home care team.* BMC Geriatr, 2014. **14**: p. 83.		✓										
	Explore validity of model with stakeholders	**Study A4** Legare, F., et al., *Validating a conceptual model for an inter-professional approach to shared decision making: a mixed methods study.* Journal of Evaluation in Clinical Practice, 2011. **17**(4): p. 554–564.			✓	✓								
B) Interprofessional shared decision making in home care: feasibility of implementation	Assess feasibility for implementation in home care services	**Study protocol B1** Legare, F., et al., *A conceptual framework for interprofessional shared decision making in home care: protocol for a feasibility study.* BMC Health Serv Res, 2011. **11**: p. 23.					✓	✓	✓	✓				
	Assess health professionals’ intention to adopt new approach	**Study B2** Legare, F., et al., *Healthcare providers’ intentions to engage in an interprofessional approach to shared decision-making in home care programs: a mixed methods study.* J Interprof Care, 2013. **27**(3): p. 214–22					✓	✓						
	Develop tool for illustrating approach for health professionals	**Study B3** Stacey, D., et al., *A systematic process for creating and appraising clinical vignettes to illustrate interprofessional shared decision making.* J Interprof Care, 2014. **28**(5): p. 453–9.					✓	✓						
	Explore family caregivers’ perceptions of approach in home care context	**Study B4** Legare, F., et al., *An interprofessional approach to shared decision making: an exploratory case study with family caregivers of one IP home care team.* BMC Geriatr, 2014. **14**: p. 83					✓							
C) Improving the decision process about location of care with the frail elderly and their caregivers	Feasibility of intervention to improve uptake of approach by home care teams & clients	**Study protocol C1** Unpublished								✓	✓	✓		
D) Implementing shared decision making in interprofessional home care teams	Implement and evaluate intervention to improve uptake of approach by home care teams and clients +/– decision aid	**Study Protocol D1** Unpublished												

### The stable period of the old paradigms

Our research team originated during a stable period characterized by shared essential elements, methods and approaches. Ten years ago two clinician scientists pursued doctoral studies in population health with a focus on shared decision-making under the supervision of a Canada Research Chair in Patient Decision Support and expert in the study of patient decision aids. In keeping with the predominant scientific approach of the time, which was to focus on shared decision-making within a single profession, one team member focused her doctoral research on shared decision-making as practised by family physicians while another investigated shared decision-making in nursing*.*

In the early 2000s, researchers in the fields of interprofessional collaboration and shared decision-making had each succeeded in demonstrating the rationale for and independent benefits of interprofessional and shared decision-making for a range of clinical and health system outcomes [25,26]. These developments were recognized by Canadian and international policymakers in various forums and government reports [3,27].

### Crises or questions that challenged the old shared decision-making paradigm

Researchers and policymakers alike began to observe in the mid-2000s that, along with many other healthcare innovations, neither effective interprofessional collaboration nor shared decision-making were being put into practice in clinical settings [14,28]. In both fields, the objects of study began to shift from explorations of the effects of interprofessional collaboration and shared decision-making to strategies for implementing them in routine clinical care. The key hypothesis formed by the research team was that efforts to implement shared decision-making in healthcare were failing in part because they did not consider the increasingly interprofessional nature of healthcare service delivery. In addition, they recognized that efforts to implement shared decision making in team settings were limited by a lack of understanding of the organizational and contextual determinants (institutional norms and values) that could influence uptake of this approach[29]. They also suspected that a process framework for implementation of shared decision-making in team contexts was lacking. The team therefore identified the need to promote shared decision-making in interprofessional care settings, and set out to advance the theoretical, conceptual and instrumental bases for doing so.

### The emergence of the new paradigm

According to Kuhn, this phase is characterized by exploration and speculation. Faced with the intractable problems of implementing shared decision-making as currently conceived, the two clinician scientists established a formal research partnership and then invited a psychologist and healthcare administrator who had participated in a Health Canada initiative to promote greater interprofessional collaboration in clinical practice. In 2007, this new interdisciplinary team initiated a research program aimed at promoting the implementation of interprofessional collaboration combined with shared decision-making, or an interprofessional shared decision-making (IP-SDM) approach, in various clinical settings. Their program had two main objectives: (a) to contribute to advancing theories, models and measures for an IP-SDM approach in primary care [30] and; (b) to develop, implement and evaluate interventions to support IP approaches to shared decision-making in various clinical settings [31]. To improve the robustness of the implementation process, they proposed a stepwise approach to implementation grounded in the Knowledge-to-Action cycle [23], a recently developed conceptual framework for knowledge translation, or for moving evidence-based knowledge into practice. The steps included: 1) knowledge syntheses to ensure a theoretical foundation for the studies, 2) evaluation of measurement tools relevant to interprofessional collaboration and shared decision-making, 3) exploration of barriers and facilitators to implementing an interprofessional shared decision-making approach, 4) the development of interventions to address barriers and facilitators to implementation, and 5) intervention studies to evaluate effectiveness and outcomes of implementation strategies in real-world clinical practice. This approach also emphasizes the involvement of knowledge users or stakeholders, including healthcare system managers and patients, in each step.

#### Conceptual challenges

While both interprofessional collaboration and shared decision-making aimed to improve quality of care, the former focussed on the relationships among health professionals whereas the latter examined the patient-provider relationship. Thus the paradigms lacked significant common elements. The emerging paradigm of a combination of the two created a “common ground”, and IP-SDM was defined as “two or more health professionals (who) collaborate with the patient in identifying best options, clarifying patient preferences and enabling patients to take more control over the treatment plan” [32].

#### Theoretical challenges

The new research team was immediately confronted with the absence of an adequate theory. Using an approach common in nursing research, they carried out a theory analysis [12] to evaluate the strengths and limitations of existing theoretical shared decision-making frameworks and their relevance to interprofessional collaboration. They identified 15 unique frameworks, none of which described in any detail how teams of health professionals shared in decision making with a patient. The theory analysis did however provide conceptual building blocks for a new interprofessional shared decision-making model, including 18 key constructs related to shared decision-making organized into four themes (shared decision-making features, participants, influencing factors, and outcomes) and ten core constructs for interprofessional collaboration. The team then organized a two-day workshop and consensus-building exercise in 2008 with researchers and healthcare professionals from various disciplines to construct an interprofessional shared decision-making model that would describe the main steps and participants in the shared decision-making process, as well as the contextual influences on IP-SDM [33]. In keeping with the Knowledge-to-Action framework, the model was subsequently validated by 79 stakeholders working at different levels of the healthcare system. Creating this model was seen as a fundamental step towards being able to communicate what the interprofessional shared decision-making approach represented and implementing the approach in practice.

#### Methodological challenges

A major methodological challenge in developing their interdisciplinary research program was moving the emerging IP-SDM knowledge into action and assessing it. Many existing measures were inappropriate for assessing an interprofessional shared decision-making approach because they were based on the conceptual bases of either interprofessional collaboration or shared decision-making, but not both. For example, most interventions used to support professional education or practice change targeted single professions as opposed to interprofessional teams, so measures use to develop and assess them were of limited value [30]. To research interprofessional shared decision-making and especially to evaluate its implementation, team members had to perform complex combinations of methodologies, and thus had to become familiar with multiple literature review methods, validation studies, mixed-methods studies, and experimental study designs (e.g. cluster randomized trials and stepped-wedge trials). Over the course of development of the research program, the team sought assistance from methodological experts in Canada and abroad, yet they still experienced many false turns along the road to completed projects. The team learned that taking their integrated knowledge translation approach, i.e. remaining faithful to the concept of involving knowledge users as active participants in the research from the very start [23], helped minimize setbacks and improved the quality and credibility of their studies.

#### Instrumental challenges

In the past decade, the team expanded the scope of its evaluation tools from assessing shared decision-making from the perspective of individual patients or providers to allowing for third-person and dyadic (taking into account the mutual influence of patient and provider) evaluations of shared decision-making. However, these more sophisticated tools were still not adequate for implementing a fully interprofessional approach to shared decision-making. The research team therefore conducted a search for shared decision-making and interprofessional collaboration tools to establish their relevance for an interprofessional shared decision-making approach. The search retrieved 24 instruments, and critical appraisal of these tools does not appear to confirm the relevance of any of them for an IP-SDM model. The team also created and validated a video-based clinical vignette to illustrate the interprofessional shared decision-making process in action [34], as well as a decision aid that reflects the key constructs of their interprofessional shared decision-making model. The vignette, showing an elderly person and a caregiver in a home care setting, has been used to demonstrate that an interprofessional shared decision-making approach is feasible in these settings, even though interprofessional involvement in the decision is asynchronous [34]. Both tools are also now components of a multifaceted IP education intervention designed to align home care practices with the new IP-SDM approach [35]. Evaluation of the intervention is still underway and the specific challenges of implementing the IP-SDM approach in real-world clinical practices (Step 5 of the implementation plan) have not yet been encountered.

### Normalization of the new paradigm and emergence of new puzzles

This stage is characterized by the expansion of knowledge in the IP-SDM field and the emergence of new puzzles that call for their own conceptual, theoretical, methodological and instrumental solutions. Normalization is often reflected in systematic reviews, new journals, textbooks or textbook chapters, granting agency requirements, as well as an increasing pool of references. In 2009, a chapter from a seminal textbook in shared decision-making focused on SDM within team-based care [36]. Similarly, in 2011, a full issue of the *Journal of Interprofessional Care* reported on the Summer Institute of Patient Choice of 2008 and was entirely dedicated to IP-SDM [25,37,38,39,40,41,42,43]. In addition, using reverse citation, we identified more than 150 unique citations and dozens of teams that have been applied this team’s approach in their own areas of work (see Table 2). Our reverse citation searches suggest an important expansion of knowledge about IP-SDM that is reaching a variety of fields, including rehabilitation [44,45], mental health [46,47], care for complex patients [48,49,50], and educational research [38,51].

**Table 2:** Reverse citation searches – notable citations [52,53].


**55 citations** of Legare, F., et al., *Advancing theories, models and measurement for an interprofessional approach to shared decision making in primary care: a study protocol.* BMC Health Serv Res, 2008. **8**(1): p. 2; including: – Elwyn et al 2009 – Decision aids and beyond – Desroches et al 2011 – SDM beliefs among dietitians – Dunn – 2011 thesis – IP-SDM in intensive care unit – Chong et al 2013 – IP and SDM in mental health care (2 articles) – Col et al 2011 – IP education and SDM – Yu et al 2014 – IP SDM and goal setting in diabetes care – Alharbi et al 2014 – Experiences of patient-centred care in medical wards – Catherine, Ivers et al 2015 – IP-SDM and decision aid for diabetes – Sieck et al – IP-SDM increasing the shared in SDM

**65 citations** of Stacey, D., et al., *Shared decision making models to inform an interprofessional perspective on decision making: a theory analysis.* Patient Educ Couns, 2010. **80**(2): p. 164–72, including: – Korner et al 2012 – IP-SDM train the trainer program – Korner et al 2013 – Designing an IP training program for SDM – Stille et al 2013 – Parent partnerships in SDM for children with special needs – Weinberger et al 2015 – IP-SDM model for the complex patient – Hajiesmaeili et al 2015 – IP-SDM approach in the intensive care unit

**89 citations** of Legare, F., et al., *Interprofessionalism and shared decision-making in primary care: a stepwise approach towards a new model.* J Interprof Care, 2011. **25**(1): p. 18–25, including: – Llewellyn-Thomas & Légaré 2011 – IP education for IP-SDM practice – Peterson 2012 – SDM in healthcare, role for social work – Hofstede et al 2012 – Implementation of IP-SDM for sciatica care – Buhse et al 2012 – SDM program for prevention of heart attack in diabetes – Ernst et al 2015 – Role of pediatric psychologists in SDM – Stacey 2015 – IP-SDM in oncology nursing

**18 citations** of Legare, F., et al., *A conceptual framework for interprofessional shared decision making in home care: protocol for a feasibility study.* BMC Health Serv Res, 2011. **11**: p. 23, including: – Truglio-Londrigan 2013 – SDM in home care from the nurses perspective – Truglio-Londrigan 2015 – Patient experience with SDM

**19 citations** of Legare, F., et al., *Healthcare providers’ intentions to engage in an interprofessional approach to shared decision-making in home care programs: a mixed methods study.* J Interprof Care, 2013. **27**(3): p. 214–22, including: – Ploeg et al 2014 – IPE and IP collaboration in home care for older adults – Sohi et al 2015 – Health care professionals’ collaboration to facilitate patient participation in life-prolonging care decisions

**4 citations** of Stacey, D., et al., *A systematic process for creating and appraising clinical vignettes to illustrate interprofessional shared decision making.* J Interprof Care, 2014. **28**(5): p. 453–9.

**2 citations** of Legare, F., et al., *An interprofessional approach to shared decision making: an exploratory case study with family caregivers of one IP home care team.* BMC Geriatr, 2014. **14**: p. 83.

Despite these advances, important puzzles remain. Methodological and instrumental questions include: 1) what are the most effective strategies for implementing an interprofessional shared decision-making approach in different clinical contexts? 2) What are the impacts of an IP-SDM approach on patient health and health system outcomes? 3) What value is added by an interprofessional approach to shared decision-making, and what are its financial and human resource implications? 4) What are the specific social, organizational, and health system factors that influence interprofessional shared decision-making processes and outcomes? and 5) How can we determine patients’ desired levels of engagement in interprofessional shared decision-making [25]? Questions relating to methodological and instrumental gaps include: 1) what are the most effective approaches to recruiting participants in order to study SDM in interprofessional contexts? 2) How do we design and validate measurement tools for measuring the practice of an IP-SDM approach that takes into consideration the various components of the model and all participants, 3) What are the strategies for collecting data on an IP-SDM approach when different members of the healthcare team are involved at different times, and team membership or involvement in decision-making changes over time? Addressing these puzzles will contribute to the further normalization of the IP-SDM research field. It is too early to say which of these puzzles will be adequately addressed using the shared concepts, theories, methods and instruments of the paradigm as it shifts into its normalization phase, and which puzzles may prove to be anomalies that could provoke a new paradigm shift. Table 3 summarizes the key puzzles in merging of interprofessional collaboration (IP) and shared decision-making (SDM) into IP-SDM.

**Table 3:** Key puzzles in merging of interprofessional collaboration (IP) and shared decision-making (SDM) into IP-SDM.

Puzzles	Type	How addressed	Opportunities

No consensus definitions of IP or SDM, therefore no definition of IP-SDM	Conceptual	IP-SDM defined	Further conceptual research that delves into both
SDM and IP lacked common elements	Conceptual	Identification of common ground by interdisciplinary research team	Further interdisciplinary collaborations that identify and build on common ground
Absence of relevant theory/model	Theoretical	Theory analysis of existing frameworks; interdisciplinary collaborative meeting to design new model; validation of model with stakeholders using Knowledge to Action framework.	Refinement of new model
Understanding the impact of the variables in each element of IP-SDM separately	Methodological	Stepwise approaches	Address complex statistical and organizational challenges of stepwise approaches
Measures only available for IP or SDM	Methodological	Learning new literature review methods, combinations of study designs	Development of new study designs and review methodologies
How to involve stakeholders effectively	Methodological	Integrated KT approach	Advancing knowledge about stakeholder involvement
How to recruit IP-SDM participants more effectively	Methodological	Managerial support, personal visits to sites; use of RAs, involvement of management & IP team from start)	Advancing knowledge about recruiting participants
Influence of social, organizational & health-system factors	Methodological	Interdisciplinary research team and a broader conceptualization of IP-SDM	Increased research on methods for measuring impact of these factors on IP-SDM needed
Determining patients’ desired levels of engagement in IP-SDM	Instrumental	Consulting patients and caregivers	Instrument development
Asynchronous nature of IP involvement, and changes in team or decision making involvement over time	Instrumental	Managerial support in involving IP teams, interdisciplinary research	Time-sensitive measures
Evaluation tools for IP-SDM	Instrumental	Search for tools; creation of video-vignette to explain approach	Identify challenges of implementation in real-world
Implementation in a variety of clinical contexts	Instrumental	Different clinical setting identified and feasibility of IP-SDM assessed	Develop strategies for implementing IP-SDM in different clinical contexts
Impacts of SDM on patient health, health systems and financial and human resources	Instrumental	Interdisciplinary research team	Advancing knowledge about these impact factors

## Discussion

According to Kuhn’s theory of scientific revolutions, our reflective synthesis suggests that the merging of the fields of interprofessional collaboration and shared decision-making may have laid the foundation for a new emergent paradigm. Our review of the team’s research documents identified both challenges to the development of an interdisciplinary interprofessional shared decision-making field that the team has not yet solved (puzzles) as well as challenges that they have successfully addressed. Challenges to which the team found solutions were the lack of conceptual clarity about an interprofessional approach to shared decision-making, a lack of theories and models about interprofessional shared decision-making, limited methodological guidance to implementing interprofessional shared decision-making, and a dearth of suitable instruments for implementing or assessing the interprofessional shared decision-making approach. The team responded to these challenges by defining an interprofessional approach to shared decision-making, developing and validating a new interprofessional shared decision-making model, grounding its research program in the Knowledge-to-Action framework, and developing tools (e.g. a clinical vignette and decision aid) to support interprofessional shared decision-making training. It has initiated efforts to implement the IP-SDM approach in real-world settings and also identified components of the model that warrant further attention, such as the need for deliberation about patients’ desired levels of engagement in the interprofessional shared decision-making process and assessing the influence of contextual factors (at the team, organization and system levels) on interprofessional shared decision-making practices.

These results lead us to make two main observations. First, our reflective process of chronicling the evolution of interdisciplinary team science over the past nine years in one research team, based on Kuhn’s theory, helped the team take a step back to identify, explain and categorize a) the challenges faced, both met and unmet, and b) the solutions this research team found to the various types of problems encountered. This gave the team a new perspective on its attempts to merge two fields, both the ground covered and the distance still to go. Individual scientists and scientific teams can become immobilized in their professional, conceptual, theoretical, methodological, and instrumental habits. Faced with end-of-paradigm anomalies related to a generalized failure to implement shared decision-making, this team’s attempt to integrate a new discipline pushed it to question its habits, innovate, and arrive at a less comfortable but more promising research horizon with implications for more pertinent clinical education and interventions.

Second, as the two fields of research connected, they became more and more entangled to the point where the two were no longer distinguishable, in keeping with the growing integrity of an emerging paradigm. This could be explained by the nature of the collaborative work of an interdisciplinary team, whose trajectory cannot be reversed without ignoring team expertise. It could also be the inevitable result of implementation science: once the concepts are producing concrete results with genuine stakeholder involvement, a momentum is established.

This reflective case study was performed by two young researchers new to the research team. It was not only timely for the research team to reflect on how the two disciplines were working together, but turned out to be an excellent capacity-building exercise for these new team members. Not only did it give them the opportunity to study the team’s productions in depth and ask questions, it also gave them a bird’s eye view of the research program and team’s evolution, its particular culture of problem-solving in four essential dimensions (conceptual, theoretical, methodological and instrumental) and a good idea of unmet challenges they might want to address in the future. In addition, it helped them develop relationships with the team, and the results of their analysis gave them a chance to make important contributions to the team’s work at an early stage of their involvement.

This reflective case study is based on the work of a single research team and as such it is unclear to what extent our experience is shared by other researchers in our field or other fields of interdisciplinary research. While a critical analysis of multiple teams might have been produced more generalizable results, our analysis of one team gave us access to unpublished protocols and opportunities for in-depth exchanges with team members who were committed to the authors on both a professional and theoretical level. As pioneers of the IP-SDM approach, team members were well-positioned to provide a detailed account of the issues emerging from the initial merging of the IP and SDM paradigms. It is our hope that insights from this analysis will resonate with and potentially inform the work of other research teams engaged in similar interdisciplinary research.

## Conclusion

In keeping with the emergence of a new paradigm, it is now a widely accepted that the delivery of safe, high-quality, and sustainable healthcare depends upon increased levels of collaboration among healthcare professionals and the engagement of patients in their own care. This is a promising new area of research that brings two distinct fields together. This emerging paradigm and its challenges, both resolved and as yet unresolved, will likely lead researchers down new and exciting paths towards a stronger, more patient-centred healthcare system.
